# Pandemic of Pregnant Obese Women: Is It Time to Re-Evaluate Antenatal Weight Loss?

**DOI:** 10.3390/healthcare3030733

**Published:** 2015-08-20

**Authors:** Anne M. Davis

**Affiliations:** Nutrition and Dietetics, University of New Haven, Boston Post Road 300, West Haven, CT 06516, USA; E-Mail: amdavis@newhaven.edu; Tel.: +1-443-986-2465

**Keywords:** obesity, pregnancy, gestational weight loss, safety, metabolism, starvation

## Abstract

The Obesity pandemic will afflict future generations without successful prevention, intervention and management. Attention to reducing obesity before, during and after pregnancy is essential for mothers and their offspring. Preconception weight loss is difficult given that many pregnancies are unplanned. Interventions aimed at limiting gestational weight gain have produced minimal maternal and infant outcomes. Therefore, increased research to develop evidence-based clinical practice is needed to adequately care for obese pregnant women especially during antenatal care. This review evaluates the current evidence of obesity interventions during pregnancy various including weight loss for safety and efficacy. Recommendations are provided with the end goal being a healthy pregnancy, optimal condition for breastfeeding and prevent the progression of obesity in future generations.

## 1. A Rising Pregnancy Health Problem

Pregnancy and infant health are commonly used to define a nation’s health and future. It also represents the quality and effectiveness of its health care and public health systems. For the past 20 years the rising obesity pandemic has received a great deal of press, policy and research but continues to elude successful remedy to date. Among women of childbearing age, obesity is paramount because of its association with multiple adverse health outcomes for both mother and fetus and even future generations [1]. When compared with other age groups, U.S. women aged 35 to 44 years have experienced the greatest increase in obesity prevalence in the past 45 years [2] and 45% of women begin pregnancy overweight or obese, up from 24% in 1983 [3]. Gestational weight gain continues to exceed the 2009 Institute of Medicine (IOM) guidelines, with 43% of pregnant women gaining more than is recommended [3]. However, considering that 22% of all global pregnancies are unplanned, current guidelines are not addressing the growing problem of being obese and becoming pregnant [4]. The total number of women who become pregnant in any given year is unknown, but approximately four million births occur in the United States annually and, currently, 60% of women of childbearing age are either overweight or obese (Table 1) [3].

**Table 1:** The international classification of adult underweight, overweight and obesity according to Body Mass Index (BMI) adapted from the World Health Organization (WHO) [5].

Classification	Principal Cut-off Points
Underweight	BMI < 18.5 kg/m^2^
Normal	BMI 18.5–24.99 kg/m^2^
Overweight/pre-obese	BMI 25–29.99 kg/m^2^
Obese class 1	BMI 30–34.99 kg/m^2^
Obese class 2	BMI 35–39.99 kg/m^2^
Obese class 3	BMI ≥ 40 kg/m^2^

There are multiple maternal complications of obesity during and after pregnancy with or without excess gestational weight gain including high blood pressure, preeclampsia, gestational diabetes, and cesarean delivery to name a few [5,6,7,8,9,10,11,12,13,14,15,16,17,18,19,20,21,22,23]. Obese pregnant women are at a higher risk of problems related to cesarean delivery—including complications with anesthesia, excessive blood loss, blood clots, and infection. Offspring are at increased risk for miscarriage, stillbirth, premature birth, or having a baby with a birth defect.

In a recent cross-sectional survey of health behaviors, including obesity risk knowledge, weight misperception, and diet and health-related attitudes among women (aged 16 to 40 years) intending to become pregnant, 51% had low obesity risk knowledge and 31% misperceived their body weight [24]. Additionally, 76% of these women felt confused about what constitutes a healthy diet, even though 47% believed that their current diet was healthy and saw no reason to change their current eating patterns. Weight misperception did not differ between the two groups, however, the overweight women intending to become pregnant were more likely to misperceive their weight than obese women intending to become pregnant (71% *vs*. 10%; *p* < 0.001).

## 2. Objective

The American College of Obstetrics and Gynecology’s (ACOG), National Institute of Health and Clinical Excellence (NICE) in the United Kingdom, Centers for Diseases Control, and the Academy of Nutrition and Dietetics’ recommendations suggest weight loss during preconception and/or limited gestational weight gain (GWG) [1,25,26,27]. The primary purpose of this paper is to examine recent data to date on hypocaloric feeding interventions to reduce maternal and neonatal complications and adverse outcomes of pregnant obese women and their fetus.

### 2.1. What History Tells Us: Recent Systematic Reviews and Meta-Analyses

A 2013 Cochrane Review, Antenatal Interventions for Reducing Weight in Obese Women for Improving Pregnancy Outcome, found no randomized controlled trials and recommended that further research is needed to evaluate the safety of interventions for weight loss when a woman is obese and becomes pregnant [28]. Types of outcome measures examined included the following primary outcomes of serious maternal morbidity (admission to high dependency care) and/or death; neonatal admission to neonatal intensive care; and perinatal death (including still birth). Secondary maternal outcomes included gestational diabetes; fetal distress in pregnancy or labor; post-partum hemorrhage; caesarean birth; infection (including wound, urinary tract, perineum, chest and breast); and weight (loss/gain/no change) [28]. Neonatal outcomes comprised of birth weight < 2500 g and < 10th percentile for gestational age and gender; birth weight > 4000 g or > 90th percentile for gestational age and gender; preterm birth (birth < 37 completed weeks of pregnancy); Apgar scores < 7 at 5 min; and hypoglycemia, as defined by researchers. Long-term outcomes were identified as maternal weight postpartum and childhood weight [28].

Subsequently, a systematic review by The Cochrane Collaboration, titled “Diet or exercise, or both, for preventing excessive weight gain in pregnancy” was aimed to determine whether diet or exercise measures, or both, could prevent excessive gestational weight gain (GWG), and if they were safe. The authors systematically analyzed 49 high quality RCTs out of a total of 65 RCTs comprising 11,444 women treated with diet or exercise, or both, or standard care [28]. Collectively, the interventions reduced the risk of excessive GWG on average by 20% overall (average risk ratio (RR) 0.80, 95% confidence interval (CI) 0.73 to 0.87). Excessive GWG is WG above the 2009 IOM pregnancy weight gain guidelines [2]. Additionally, women receiving diet or exercise, or both interventions were more likely to experience low GWG than those in control groups (average RR 1.14, 95% CI 1.02 to 1.27); maternal hypertension (not a pre-specified outcome) was reduced in the intervention group compared with the control group overall (average RR 0.70, 95% CI 0.51 to 0.96) and a 13% (−1% to 25%) reduction for combined diet and exercise counselling interventions. However, no differences were found between intervention and control groups with regard to pre-eclampsia (RR 0.95, 95% CI 0.77 to 1.16); cesarean delivery overall (RR 0.95, 95% CI 0.88 to 1.03); preterm birth overall (average RR 0.91, 95% CI 0.68 to 1.22); no clear difference between intervention and control groups with regard to infant macrosomia (average RR 0.93, 95% CI 0.86 to 1.02); and no differences in the risk of poor neonatal outcomes including shoulder dystocia, neonatal hypoglycemia, hyperbilirubinemia, or birth trauma (all moderate-quality evidence) between intervention and control groups; however, infants of high-risk women had a reduced risk of respiratory distress syndrome if their mothers were in the intervention group (RR 0.47, 95% CI 0.26 to 0.85) [29].

Thangaratinam and colleagues in a systematic review and meta-analysis evaluated 44 RCTs (7278 women) consisting of three categories of interventions: diet, physical activity, and a mixed method [30]. Results showed a 1.42 kg reduction (95% confidence interval 0.95 to 1.89 kg) in gestational weight gain with any intervention compared to control. When combining all interventions, there were no significant differences in birth weight (mean difference −50 g, −100 to 0 g) and the incidence of large for gestational age (relative risk 0.85, 0.66 to 1.09) or small for gestational age (relative risk 1.00, 0.78 to 1.28) babies between the groups, though by itself physical activity was associated with reduced birth weight (mean difference −60 g, −120 to −10 g). Interventions were associated with a reduced the risk of preeclampsia (relative risk 0.74, 0.60 to 0.92) and shoulder dystocia (relative risk 0.39, 0.22 to 0.70) [30].

Another systematic review and meta-analysis on restriction of gestational weight gain conducted by Quinlivan and colleagues found four RCTs comprised of 537 women that met the inclusion/exclusion criteria [31]. The intervention programs were effective in reducing the total gestational weight gain by 6.5 kg. In spite of this, antenatal dietary interventions did not alter newborn birth weight (z = 0.18, *p* = 0.859).

More recently, a pilot RCT comprised of a multifaceted lifestyle (diet and exercise) treatment in obese pregnant women (the UPBEAT trial) found that baseline energy intake decreased from 1734 kcal/d to 1612 kcal/d (a 7% decrease over seven months) [31]. Treatment dietary advice comprised of increasing low glycemic index (GI) carbohydrates, replacing sugar sweetened beverages with low GI replacements and replacing foods high in low saturated fat with polyunsaturated and monounsaturated fat alternatives. Physical activity advice encourage walking at a moderate intensity. Limiting energy intake was not recommended. Outcome data collected but not reported included diagnosis of GDM and gestational weight gain. Unfortunately, neither ketonuria nor ketonemia was measured.

### 2.2. Non-Randomized Controlled Trials

Little data exists on the effects of weight loss on fetal growth [32]. Other investigations and authors suggest rates of preeclampsia, caesarean delivery, and other adverse outcomes are reduced when weight gain is limited to seven kilograms [33,34]. Lower GWG and no GWG has been shown to not be harmful, but what about actual weight loss during pregnancy? Weight gain restriction during pregnancy is safe for both the mother and neonate [35,36]. When assessing weight gain restriction studies, it is difficult to determine the level of energy/macronutrient/micronutrient restriction used and whether metabolic monitoring was applied through adverse event reporting.

A retrospective cohort of study of 1344 women with GDM evaluated the association of obesity with pregnancy outcomes in women via a medical record review [37]. Findings included higher mean fasting and postprandial glucose levels despite medication in the overweight and obese women. Obesity was associated with macrosomia (adjusted odds ratio [OR] 2.03, 95% confidence interval [CI] 1.07–3.89, *p* = 0.03), preterm birth (adjusted OR 2.21, 95% CI 1.02–4.78, *p* = 0.04), and hypertensive disorders of pregnancy (adjusted OR 2.19, 95% CI 1.38–3.49, *p* = 0.001). Obese women with fasting blood sugars greater than 88.7 mg/dL and postprandial blood sugars greater than 123.8 mg/dL. Increased blood pressure problems were significantly increased in obese women when gestational weight gain was >0.6 lb. per week (29.4% compared with 15.2%, *p* < 0.001) as compared to obese women with less weight gain [37].

Emerging evidence suggests that weight loss in pregnancy for obese women may have substantial benefits for both the mother and infant. In 2009, Oken and colleagues reviewed 2011 mother-child pairs against five adverse outcomes related to gestational weight gain: preterm birth, small-for-gestational-age infant, large-for-gestational-age infant, substantial maternal postpartum weight retention, and child obesity at age of three years. The results indicated that the lowest predicted prevalence of all five adverse outcomes occurred with a weight loss of 0.19 kg/week for obese women, which equates to a total loss of 7.6 kg for obese women over all of the pregnancy [30,38].

### 2.3. Metabolism during Pregnancy Compounded by Obesity

Adipose tissue works as an endocrine organ and it is the metabolic function of adipose tissue that causes much of the pathology associated with obesity [39]. It stores and secretes preformed steroid hormones, converts precursors to biologically active hormones, and converts active hormones to inactive metabolites. Adipose tissue secretes enzymes needed for steroid hormone biosynthesis and metabolism, such as estrone, which is converted to estradiol in peripheral adipose tissue. Adipose tissue expresses 11-β-hydroxysteroid dehydrogenase type 1 (11-β-HSD1), which converts cortisone to cortisol, as well as 5-α-reductase, which converts cortisol to 5-α-tetrahydrocortisol. Thus, adipose tissue regulates the local concentration of glucocorticoids and contributes to their metabolic clearance and secretes a large number of bioactive peptides and cytokines, (adipokines) (Table 2). The metabolic consequences of obesity are similar to the endocrine dysfunction seen in hyperplasia of any endocrine organ.

**Table 2:** Enzymes and hormones produced by adipose tissue [39].

Enzyme/Hormone	Function	Changes Associated with Obesity
Aromatase	Converts androgens to estrogens	No change with obesity, but increased fat mass results in greater total conversion
17-β-hydroxysteroid hydrogenase	Converts estrone to estradiol and androstendione to testosterone	No change
5-α-reductase	Inactivates cortisol	No change
11-β-hydroxysteroid dehydrogenase type 1	Converts cortisone to cortisol	Activity is increased in obese women
Leptin	Affects food intake, timing of puberty, bone development, and immune function	Circulating leptin levels are increased in obese women
Tumor necrosis α factor (TNFα)	Represses genes involved in the uptake and storage of nonesterfied fatty acids and glucose	Expression of TNFα is increased in the adipose tissue of obese women
Adiponectin	Enhances insulin action	Circulating levels of adiponectin are decreased in obese women

Carbohydrate (CHO) metabolism in pregnancy is characterized by mild fasting hypoglycemia, post-prandial hyperglycemia and hyperinsulinemia. In pregnant women, after CHO ingestion, there is prolonged hyperglycemia and hyperinsulinemia, as well as increased suppression of glucagon secretion [40]. This response is related to the induced state of peripheral insulin resistance (IR), which is even more pronounced in obese pregnant women and gestational diabetes [41]. The purpose of IR is thought to maintain a postprandial supply of glucose to the fetus as the fetus is estimated to utilize 20–25 g of glucose a day in late pregnancy [42].

Decreased insulin sensitivity during pregnancy is also related to lipid metabolism [43]. Increased concentrations of free fatty acids are related to decreased insulin ability to suppress lipolysis in late pregnancy. Freinkel used the term “accelerated starvation in pregnancy” to describe the increased risk of ketosis in pregnant women [44]. Lipid metabolism in obese women and in lean women experience the predominance of lipogenesis in the first trimester of pregnancy, and lipolysis in the third trimester. In obese women, lipogenesis occurs only in the pre-pregnancy period, whereas lipolysis predominates in all trimesters of pregnancy [45]. These data confirm insulin’s inability to suppress lipolysis in all women as pregnancy advances, and further confirm the evidence of increased IR in obese women, as compared to women with normal weight in earlier stages of pregnancy [43]. Obese pregnant women exhibit a more pronounced hyperlipidemia and the adipocytes and adipose stromal cells are a rich source of cytokines and inflammatory mediators, which can either increase IR (TNF-α), or reduce it (adiponectin).

Contrary to expectations, there is no increase in measured protein synthesis in the first trimester, there is a 15% increase in the second trimester and a 25% increase in the third trimester [46]. The concentration of amino acids is higher in the fetus than the mother [47,48] and during pregnancy, most amino acids are used for protein synthesis, with a decrease in their oxidation by approximately 10% [49]. Presently, the effects of obesity on amino acid metabolism are unknown. Because hyperinsulinemia decreases protein synthesis in non-pregnant obese women, there may be a possibility of impaired anabolic response to pregnancy and a mechanism limiting fetal growth.

## 3. Weight Loss during Pregnancy

History provides observations and studies of extreme states of weight loss and dieting during pregnancy in comorbidities such as hyperemesis gravidarum and malnutrition, starvation and the Dutch Famine and the hormonal effects of fasting during pregnancy. Although this data does not specifically address additional exposure to the state of obesity, these three conditions assess safety and maternal and fetal outcomes of nutritional deprivation during pregnancy.

In starvation, suppression of the sympathetic nervous system likely contributes to a decrease in metabolic rate with energy restriction and together the stimulation of the adreno-medullary system allows for substrate mobilization with a minimal increase in energy expenditure [49]. In addition, starvation drastically reduces the amount of amino acids in the amniotic fluid [50]. However, it is still unclear when and how much deprivation is needed to influence pregnancy outcome positively and negatively.

### 3.1. The Dutch Famine

The Dutch famine in 1944–1945 exemplifies the effect of timing of poor nutrition on low birth weight [51]. Third trimester exposure accounted for the whole of the famine effects on birth weight whereas first trimester exposure affected the length of gestation. Of particular note, the first trimester pregnant woman is considered to be more vulnerable to stress and during the famine the stress of war may have been the stressor affecting gestational length but not birth weight.

Offspring whose mothers had been exposed to famine in the second or third trimester had reduced glucose tolerance, shown by increased two-hour plasma glucose concentrations [52]. Those individuals who had smaller birth weights had increased two-hour plasma glucose concentrations. Children exposed to famine during the first trimester had a more atherogenic lipid profile [53], somewhat higher fibrinogen concentrations and reduced plasma concentrations of factor VII [54], a higher BMI [55] and they appeared to have a higher risk of CHD [51,56]. Offspring whose mothers consumed little protein relative to carbohydrate during the third trimester of pregnancy had higher blood pressures at adult age [57]. The implication is that blood pressure may be linked to a disruption in the balance of macronutrients in the maternal diet during late gestation rather than to absolute amounts of nutrients.

Recognized infant outcomes of low birth weight, still birth and congenital anomalies from associated intrauterine exposure to famine have been associated with adult diabetes, obesity cardiovascular disease schizophrenia, cognitive aging, hypomethylation of IGF2 and INSIGF hypermethylation of ABCA1, GNASAS, IL10, LEP, and MEG3 stochastic methylation changes of several metastable epialleles [58,59]. Much of these long-term effects are from observations from the Dutch Hunger Winter Study and the discovery of the significance of timing in the fetal programming of adult disease. Those who were exposed to the famine only during late gestation were born small and continued to be small throughout their lives, with lower rates of obesity as adults than in those born before and after the famine [60]. Those offspring exposed during early gestation experienced elevated rates of obesity, altered lipid profiles, and cardiovascular disease. Additionally, those exposed in mid-pregnancy demonstrated reduced renal function [61,62]. Those exposed to famine during early gestation later showed a significant impairment in a test of selective attention at age 56–59 years [63]. These observations represented first critical windows of development preceding the developmental origins of health disease hypothesis [63]. Moderate nutritional deficiency experienced by the pregnant women of the Dutch Famine was considered to be a mean daily ration of 1000 to 1500 calories per day with estimated intakes of 37 to 42 g of protein, 212 to 247 g CHO and 24 to 41 g of fat [55]. Severe nutritional deficiency exposure was judged to be a mean daily ration of <1000 calories per day, 14 to 22 g of protein, 114 to 144 g of CHO and 12 to 28 g of fat (Table 3) [55]. At the beginning of the famine, wartime maternal weights were low in comparison with the immediate postwar norm [55]. Maternal weight quickly dropped following a 39.4% reduction in food rations. From September 1944 through April 1945, daily food rations changed from a high of 1924 calories to a low of 836 calories (Table 2) [55]. The average decline from the pre-famine level in the most affected cohort was 2.6 kg, or 4.3%, and the rise with recovery was 5.9 kg, or 10.5% [55].

**Table 3:** The Dutch Famine daily food rations by week and pregnancy trimester periods July 1944 to July 1945.

Time Period	Calories	Protein	Carbohydrate	Fat
September 1944	1924 (64%)	61 (73%)	295 (68%)	50 (42%)
February 1945	836 (28%)	35 (42%)	136 (31%)	16 (14%)
April 1945	862 (29%)	35 (41%)	144 (33%)	14 (14%)
Pregnancy Reference§	3000	84	432	102

§ Reference: Oxford Nutrition Survey Standards. (Source: Dolsand van Arckeren 1946, p 358) [55]; (% Percentage) represents adequacy of intake compared to reference standard.

### 3.2. Fasting

Hormones derived from the placenta including glucagon, cortisol, and human placental lactogen create the insulin deficient state in pregnancy and are further increased during periods of stress, such as fasting and starvation [61]. During fasting, circulating glucose levels fall, leading to a decrease in insulin secretion. At the same time, levels of glucagon and catecholamines resulting in the breakdown of glycogen, and gluconeogenesis is increased. As fasting continues for more than several hours, glycogen stores become depleted, and the low levels of circulating insulin promote increased fatty acid release from adipocytes. Oxidation of fatty acids generates ketones that can be used as fuel by skeletal and cardiac muscle, liver, kidney, and adipose tissue, thus sparing glucose for continued utilization by brain and erythrocytes [64].

Siega-Riz and colleagues reported that meal patterns of pregnant women and the frequency of food intake during pregnancy are relevant to the relationship between maternal nutrition status and preterm birth [65]. Risk factors included consuming fewer than three meals and two snacks per day had a 30% higher risk for delivering preterm and not eating for >13 h per day had a three-fold greater risk of delivering preterm at <34 weeks gestation. Metzger and Felig from two separate studies found that a 16-hour fast in a pregnant group resulted in significantly lower glucose levels and significantly higher free fatty acids and β-hydroxybutyrate levels in both lean and obese women [66,67].

Another condition of religious fasting during the holy month of Ramadan, may pose risks to pregnant women. In a sample of 30 fasting pregnant women, Khoshdel and colleagues measured significant changes in follicle stimulating hormone (FSH), estrogen, progesterone and leptin levels (*p* < 0.05) [68]. Additionally, poor weight gain in the pregnant fasted women was observed during the study [68]. In another study, Ziaee and colleagues studied a historical cohort of pregnant women seen at Tehran hospitals at one of the trimesters in the holy month of Ramadan [69]. The women were divided into non-fasting, 11–20 days fasting, and 21–30 days fasting. There was a relative risk of low birth weight incidence in mothers fasting during the first trimester compared to the non-fasting mothers [69]. An increased risk of hyperemesis gravidarum in Ramadan fasting women during the first month of pregnancy has been found [69].

Herrmann and colleagues found that periods without food lasting 13 h during pregnancy compared with periods <13 h were associated with elevated maternal corticotrophin-releasing hormone (CRH) concentrations after controlling for pre-pregnancy BMI energy intake, income, race, smoking, and maternal age [70]. This was the first time a plausible mechanism, placental CRH, for the relationship between fasting and a greater risk of preterm birth was identified. Some studies suggest that severe maternal acidosis has an adverse maternal and fetal impact including fetal neurological impairment and fetal loss. Similar metabolic changes are seen with poor dietary intake or prolonged fasting and resulting acidosis is referred to as “starvation ketoacidosis” [64].

### 3.3. Hyperemesis Gravidarum

The Norwegian Mother and Child Cohort Study comprising of 108,000 births was the setting used to explore relationships between hyperemesis gravidarum (HG) and adverse pregnancy outcomes [71]. Characteristics of the pregnant women in the cohort who had HG (1.1%) included younger age, less education, and were mainly non-smokers before pregnancy. They had lower or higher pre-pregnancy BMI compared to those without HG. Additionally, a majority of the women with HG gained less weight during pregnancy than those without HG. Women with HG gained on average 12.7 kg during pregnancy, compared to 14.9 kg for women without HG [72]. Other complications reported via case studies are maternal metabolic alkalosis and neonatal metabolic acidosis [73,74]. In a sample of 33,467 primiparous women from the Norwegian Mother and Child Cohort Study (1999−2008), 353 (1.1%) women had hyperemesis. Among non-smokers, both underweight and obese women were more likely to develop hyperemesis than normal-weighted women: odds ratio (OR), 2.36; 95% confidence interval (95% CI), 1.43−3.88 and OR, 1.48; 95% CI, 1.00−2.20, respectively [75]. When comparing the results of this cohort to other studies, HG was not found to be associated with an increased risk of preterm birth, low birth weight and small for gestational age [76,77]. Conflicting results may have been due to the use of different HG diagnostic criteria since a universally accepted definition is lacking. Some definitions included dehydration, electrolyte disturbances, ketonuria and more than 5% weight loss compared to pre-pregnancy weight whereas the Norwegian cohort defined HG as long-lasting nausea and vomiting in pregnancy starting before the 25th gestational week, which required hospitalization [72]. Hyperemesis gravidarum can be extremely debilitating for women and, if inadequately managed, can cause significant morbidities, including malnutrition and electrolyte imbalances, thrombosis, Wernicke’s encephalopathy, depressive illness, and poor pregnancy outcomes, such as prematurity and small-for-gestational-age fetuses. [78,79,80,81]

### 3.4. Eating Disorders in Pregnancy

Even less is known about metabolic disturbances in normal weight and obese women during pregnancy who either have an active eating disorder (ED) or have a history of one—anorexia nervosa (AN), bulimia nervosa (BN), binge-eating disorder (BED) and eating disorders not otherwise specified (EDNOS) [82,83]. Poor nutrition to the fetus is known to negatively impact brain development and increase the risk of neuropsychiatric diseases [83,84]. Glucose homeostasis is likely disrupted in pregnant women with ED and both symptomatic and asymptomatic hypoglycemia can occur. Women with ED may have lowered blood glucose and insulin and may show reactive hypoglycemia after an oral glucose tolerance test [85]. Binging on foods with a high glycemic index can lead to a rapid increase in blood glucose level. When this is followed by vomiting, disturbance of the complex interaction between gut hormones and digestion occurs, resulting in rapid drops in blood glucose and leading to rapid fluctuations in blood glucose [85,86].

## 4. Weight Loss Diets for Obese Pregnant Women and Gestational Diabetes

There are a few energy/protein restricted studies in pregnant women with overweight/obesity or high gestational weight gain [86]. Three trials, involving 384 women, were included. Both Campbell trials reported that energy/protein restriction was associated with a significant reduction in weekly maternal weight gain, although the magnitude of the reduction was much larger in the 1975 trial of Aberdeen adults (random effects weighted mean difference (WMD) −254.81 (95% CI –436.56 to −73.06) g/week) (Table 4) [87,88]. The 1983 Campbell study found different effects of the mother’s carbohydrate intake on blood pressure (BP), depending on the level of animal protein in the diet, with low-carbohydrate, high-protein intakes related to reduced placental size and increased BP later in life [88]. Energy/protein restriction had no effect on either (proteinuric) pre-eclampsia or pregnancy-induced hypertension (with or without proteinuria), although the small number of trials and participants provides inadequate statistical power to exclude a small effect. The two trials that reported on birth weight (Badrawi 1993; Campbell 1983) yielded highly (and statistically significantly) heterogeneous results, with the Campbell 1983 study reporting basically no effect of the intervention (WMD 6.00, 95% CI −121.55 to +133.55, g) but the 1993 Badrawi study found a large significant adverse effect (WMD −450.00 g (95% CI −624.72 to −275.28) [88,89]. These large differences in results could reflect either differences in study samples (Scotland *vs*. Egypt) or differences in the degree of energy/protein restriction achieved. Only the 1983 Campbell study reported results bearing on gestational duration; the results appear to exclude an important adverse effect of dietary restriction (WMD in mean gestational age = +0.25 (−0.17 to +0.67) week) [89]. Other outcomes, including fetal/infant mortality and other measures of maternal morbidity (e.g., cesarean section) or postpartum weight retention, were not reported [90,91,92,93,94]. The fetal effects from hyperketonemia are a major concern by inducing intentional maternal weight loss during pregnancy in obese women. The presence of ketosis implies two things: (1) that lipid energy metabolism has been activated and (2) that the entire pathway of lipolysis is intact. Ketosis is normal during fasting, and is particularly important for the brain, which has no other substantial non-glucose-derived energy source. During the neonatal period, infancy, and pregnancy, times at which lipid energy metabolism is particularly active, ketosis develops readily [91]. Magee and colleagues conducted a RCT in a metabolic unit to understand the metabolic effects of a 1200-calorie diet on obese pregnant women with gestational diabetes [92]. Maternal glycemic status improved in the calorie-restricted group however there were significant increases in ketonemia and ketonuria as well. Available research to date does still not explain how hyperketonemia affects fetal metabolism, development, and overall safety.

**Table 4:** Studies using hypocaloric antenatal diets for obese pregnancy and gestational diabetes.

Author	Sample	Dietary Treatment	Outcome(s)
**Obese Pregnancy**			
Badrawi 1993 [89]	100 obese multiparous Egyptian women age 25–35 years.	Treatment: balanced low-energy (1500–2000 kcal/day) diet. Control: normal diet according to WHO energy recommendations (2300–3000 kcal/day).	Gestational weight gain, Birth weight, and PIH
Campbell 1975 [87]	153 primiparous Scottish women with high gestational weight gain (>1.25 lb. or 570 g per week) between 20 and 30 weeks.	Intervention: low-energy (1200 kcal/day), low-carbohydrate diet beginning at 30 weeks. Control: no intervention.	Gestational weight gain, PIH, and pre-eclampsia
Campbell 1983 [88]	182 obese (>75th centile weight-for-height) Scottish primiparous women with normal IVGTT	Intervention: low-energy (1250 kcal/day) diet. Control: no intervention.	Gestational weight gain, birth weight, birth length at 28 weeks gestational age, preterm birth, pre-eclampsia.
**Gestational Diabetes (GDM)**			
Knopp 1991 [90]	12 overweight GDM	1200 kcal (50% restriction) *vs*. 2400 kcal 150 g (50%) *vs*. 300 g CHO/d (50%)	1200 kcal improved (randomized) glucose; elevated ketones
Knopp 1991 [91]	6 overweight GDM (randomized)	1600–1800 (30%–33% restriction) *vs*. 2500 kcal plus prophylactic insulin; 200 g (50%) *vs*. 300 g CHO/d (50%)	1600–1800 kcal restriction improved glucose and trig with no marked ketonuria
Algert 1985 [94]	22 obese (non-randomized)	1700–1800 kcal; 212–225 g CHO/d (50%–60%)	Lower weight gain, higher; mean birth weight, no ketonuria
Magee 1990 [92]	12 obese (randomized)	1200 kcal (50% restriction) *vs*. 2400 kcal (usual intake); 150 g (50%) *vs*. 300 g CHO/d) (50%)	1.200 kcal lowered mean glucose, no change in fasting plasma glucose, Increased ketonemia
Rae 2000 [93]	66 intervention *vs*. control with insulin (randomized)	1590–1776 kcal (30% restriction) *vs*. 2010–2220 kcal; 210–244 g (51%) *vs*. 240–274 g CHO/d (46%)	No difference in frequency of insulin use; lower kcal had lower dose; no increase in ketones

Total of 5 studies: N = 553 subjects.

## 5. Beyond Necessary Research

Ideally, weight loss should occur before pregnancy, however in 2006, 49% of pregnancies in the United States were unintended—a slight increase from 48% in 2001 [95]. Realistically, significant weight loss is unlikely to happen before pregnancy with the exception of bariatric surgery. Larger RCTs are needed with specific attention to safety. Recruitment may be difficult as seen in other trials due to the stigma associated with obesity and the sensitivity (or lack of) from health care professionals who treat obese patients [36]. Delicate training of study staff will improve compassion especially with study interactions and the use of large anthropometric equipment used to weigh and measure obese subjects.

Women with polycystic ovary syndrome (PCOS) who become pregnant and classified as obese include a specific population who would benefit from weight loss research to prevent GDM and other maternal and infant complications. Weight loss for women with PCOS helps reduce the male hormone levels, obesity, and some women will begin to ovulate naturally. The effects of weight loss on hormone levels (estrogen, FSH, LH, testosterone and 17-ketosteroids) are unknown for obese pregnant women with PCOS [96]. Other unknown metabolic consequences of intentional weight loss in obese pregnant women in need of further investigation include hyperinsulinemia effects on protein synthesis, optimal energy restriction, and rate of weight loss exclusive of maternal and fetal adverse outcomes, amino acid decrease in amniotic fluid, safest meal spacing, ideal monitoring parameters, and increased ketones to name a few [97].

More investigation about defining a minimum adequate diet for pregnant women is critical for safe weight loss for maternal and fetal health. Long-term effects of weight loss diets during pregnancy without starvation will stipulate future cohorts to monitor the development of degenerative and chronic disease [98].

## 6. Conclusions

This paper highlights the possibility that safe weight loss during pregnancy for obese women is an opportunity for further investigation. It appears from available weight loss studies that 1600–1700 kcal per day does not produce ketonuria as long as 50% of the calories are provided with carbohydrate, and adequate protein, fat and micronutrients for pregnancy are supplied. Frequent monitoring during intentional pregnancy weight loss should include indirect calorimetry, serum ketones and fasting blood glucose and at a minimum.
